# Review of the spore-feeding Idolothripinae from China (Thysanoptera, Phlaeothripidae)

**DOI:** 10.3897/zookeys.345.6167

**Published:** 2013-10-29

**Authors:** Li-Hong Dang, Ge-Xia Qiao

**Affiliations:** 1 Key Laboratory of Zoological Systematics and Evolution, Institute of Zoology, Chinese Academy of Sciences, No. 1 Beichen West Road, Chaoyang District, Beijing 100101, P.R.China; 2 University of Chinese Academy of Sciences, No. 19, Yuquan Road, Shijingshan District, Beijing 100049, P.R.China

**Keywords:** Idolothripinae, genera, illustrated keys, species checklist, new records, China

## Abstract

An illustrated key is provided to the 19 genera of the subfamily Idolothripinae from China, and a checklist given to 62 named species, of which six species are newly recorded from China, together with the genus *Bolothrips* that is represented by two un-named species. A generic diagnosis is given for each genus, along with some discussion of systematic relationship problems and species diversity. Identification keys to species of 11 genera are provided, and *Megathrips antennatus* Guo, Feng & Duan is considered as a new synonymof *Megathrips lativentris* (Heeger).

## Introduction

The insect order Thysanoptera, comprises more than 6000 species, and is classified into two suborders, Terebrantia and Tubulifera (ThripsWiki 2013). There are 14 families in the two suborders, of which five families are known only from fossil species. The suborder Tubulifera includes only one family, the Phlaeothripidae, and is the largest Thysanoptera family with nearly 3550 species in 460 genera. Adults in this family have the tenth abdominal segment tubular, and the species are grouped into two subfamilies, the Idolothripinae and Phlaeothripinae. Currently there are 81 genera and 722 species listed worldwide in the Idolothripinae (ThripsWiki 2013). Species in this subfamily have broad maxillary stylets that are more than five microns in diameter, and apparently feed on fungal spores. Mound and Palmer (1983) revised and provided a key to the world genera of Idolothripinae, but the genera from China sometimes do not fit easily into this key. Okajima (2006) provided a key to 17 genera of Idolothripinae from Japan, and Eow et al. (2011) provided a key to 31 genera of Idolothripinae from Southeast Asia. Both of these recent keys are useful when studying Idolothripinae from Southern China. However, environments within China are highly varied, with considerable floristic and faunistic differences between the temperate Palaearctic north and the southern tropical and subtropical Oriental Region. Therefore a new key to the 19 genera known from Chinese territory is presented here, particularly for the convenience of students in this country.

The present work, as part of ongoing studies on the Phlaeothripidae from China, aims at providing an identification key to the 19 genera and 62 species of Idolothripinae now recorded from China. A species checklist is included, with distributional information based on the provinces and autonomous regions of China (Table 1), and a diagnosis is provided for each genus. The genus *Bolothrips* is here newly recorded from China, and six species of Idolothripinae are also newly recorded (Table 1). Moreover, *Megathrips antennatus* Guo, Feng & Duan is here considered as a new synonym of *Megathrips lativentris* (Heeger).

**Table 1.** Idolothripinae recorded from China.

**Taxa**	**Distribution in China by Provinces or Autonomous Regions**
*Acallurothrips casuarinae* Okajima, 1993	Taiwan
*Acallurothrips hagai* Okajima, 1993	Taiwan
*Acallurothrips nonakai* Okajima, 1993	Taiwan
*Acallurothrips tubullatus* Wang & Tong, 2008	Guangdong
*Allothrips bicolor* Ananthakrishnan, 1964	Hainan
*Allothrips discolor* Chen, 1982	Taiwan
*Allothrips taiwanus* Okajima, 1987	Taiwan
*Bactrothrips brevitubus* Takahashi,1935	Southern China
*Bactrothrips elongates* Dang & Qiao, 2012	Guangxi
*Bactrothrips flectoventris* Haga & Okajima, 1989	Hainan
*Bactrothrips furvescrus* Dang & Qiao, 2012	Zhejiang
*Bactrothrips honoris* (Bagnall, 1921)	Guangxi; Yunnan
*Bactrothrips pictipes* Haga & Okajima, 1989	Tibet; Guangxi; Hainan
*Bactrothrips quadrituberculatus* (Bagnall, 1908)	Yunnan; Hainan
§*Bolothrips* spp.	Beijing; Inner Mongolia
*Compsothrips reticulates* Guo & Feng, 2006	Hebei
*Compsothrips sinensis* (Pelikan, 1961)	Guangdong
*Compsothrips tenebronus* (Han & Cui, 1991)	Sichuan
*Cryptothrips nigripes* (Reuter, 1880)	Inner Mongolia
*Cryptothrips sauteri* Karny, 1913	Taiwan [known only from description]
*Dinothrips hainanensis* Zhang, 1982	Guangdong; Hainan
*Dinothrips juglandis* Moulton, 1933	Tibet; Guangdong
*Dinothrips spinosus* (Schmutz, 1913)	Yunnan; Hainan
*Dinothrips sumatrensis* Bagnall, 1908	Fujian; Guangdong; Hainan
*Dinothrips* sp.	Yunnan
*Elaphrothrips denticollis* (Bagnall, 1909)	Southern China
§*Elaphrothrips fulmeki* Priesner, 1935	Hainan
*Elaphrothrips greeni* (Bagnall, 1914)	Fujian; Yunnan; Hainan; Tibet
§*Elaphrothrips insignis* Ananthakrishnan, 1973	Yunnan; Hubei
*Elaphrothrips jacobsoni* Priesner, 1935	Guizhou
§*Elaphrothrips malayensis* (Bagnall, 1909)	Hubei; Guizhou; Yunnan; Hainan; Tibet
§*Elaphrothrips procer* (Schmutz, 1913)	Hubei; Yunnan; Guangdong; Hainan
*Elaphrothrips spiniceps* Bagnall, 1932	Fujian; Guangxi; Guangdong; Yunnan; Hainan; Taiwan
*Ethirothrips chui* (Chen, 1982)	Taiwan
*Ethirothrips longisetis* Ananthakrishnan & Jagadish, 1970	Hainan
*Ethirothrips stenomelas* (Walker, 1859)	Guangdong; Hainan
§§*Ethirothrips virgulae* (Chen, 1980)	Guangxi; Taiwan
*Ethirothrips vitreipennis* (Priesner, 1939)	Hainan
*Gastrothrips eurypelta* Cao et al., 2009	Hebei; Shaanxi; Shanxi
*Gastrothrips fulvipes* Hood, 1973	Taiwan
*Gastrothrips fuscatus* Okajima, 1979	Guangdong; Taiwan
*Gastrothrips mongolicus* (Pelikan, 1965)	Inner Mongolia; Fujian; Zhejiang; Sichuan; Ningxia
§*Gastrothrips monticola* Okajima, 2006	Inner Mongolia
*Holurothrips morikawai* Kurosawa, 1968	Guangdong; Fujian; Hainan; Taiwan
*Machatothrips antennatus* (Bagnall, 1915)	Guangdong; Hainan
*Machatothrips artocarpi* Moulton, 1928	Hainan; Taiwan
*Machatothrips celosia* Moulton, 1928	Hainan; Taiwan
*Mecynothrips kanoi* (Takahashi, 1937)	Taiwan
*Mecynothrips pugilator* (Karny, 1913)	Taiwan
§*Mecynothrips simplex* Bagnall, 1912	Yunnan; Hainan
*Mecynothrips taiwanus* Okajima, 1979	Yunnan; Hainan; Taiwan
*Megalothrips roundus* Guo et al., 2010	Hubei
*Megathrips lativentris* (Heeger, 1852)	Hebei; Henan
*Meiothrips fuscicrus* Dang & Qiao, 2012	Yunnan
*Meiothrips menoni* Ananthakrishnan, 1964	Yunnan; Hainan
*Meiothrips nepalensis* Kudo & Ananthakrishnan, 1974	Yunnan
*Nesothrips brevicollis* (Bagnall, 1914)	Henan; Shaanxi; Gansu; Taiwan
*Nesothrips lativentris* (Karny, 1913)	Guangxi; Taiwan
*Nesothrips peltatus* Han, 1991	Sichuan
*Ophthalmothrips formosanus* (Karny, 1913)	Henan; Taiwan
*Ophthalmothrips longiceps* (Haga, 1975)	Hainan; Taiwan
*Ophthalmothrips miscanthicola* (Haga, 1975)	Fujian; Guangdong; Sichuan; Hainan
*Ophthalmothrips yunnanensis* Cao et al., 2010	Yunnan
*Phaulothrips solifer* Okajima, 1989	Taiwan

§ Newly recorded from China;

§§ Newly recorded from mainland China.

## Systematics of Idolothripinae

Mound and Palmer (1983) recognized two tribes within the Idolothripinae. The Chinese fauna includes nine genera that represent the tribe Idolothripini, and 10 genera that represent the Pygothripini. In species of Idolothripini the abdominal tergites usually bear two or more pairs of wing-retaining setae, the metathoracic sternopleural sutures are never developed, and the tube is relatively long and sometimes bears long lateral setae. Species of Pygothripini, in contrast, only have one pair of wing-retaining setae on each tergite, the metathoracic sternopleural sutures are present or absent, and the tube does not have long lateral setae. Within the Pygothripini, six subtribes are recognized, and these are represented in China as follows: Pygothripina (*Cryptothrips*; *Phaulothrips*), Allothripina (*Allothrips*), Compsothripina (*Bolothrips*; *Compsothrips*), Gastrothripina (*Gastrothrips*), Diceratothripina (*Acallurothrips*; *Nesothrips*), and Macrothripina (*Ethirothrips*; *Machatothrips*). Within the Idolothripini three subtribes are recognised. The Hystricothripina occurs mainly in the Neotropics, but one genus, *Holurothrips*, is found in China. The Elaphrothripina occurs throughout the tropics, and the following genera are recorded from China, *Dinothrips*, *Elaphrothrips*, *Mecynothrips*, and *Ophthalmothrips*. The Idolothripina occurs mainly in the Palaeotropics and Palaearctic regions, and in China includes the following four genera, *Bactrothrips*, *Megalothrips*, *Megathrips* and *Meiothrips*.

### Idolothripinae fauna of China

In the only available review of the Thysanoptera fauna of China, Han (1997) provided an identification key to nine genera and 16 species of Idolothripinae known at that time from China. Unfortunately, the nomenclature and generic concepts in that study are now out-of-date, and many more taxa have been added in recent years by various authors from China and Japan. As a result, Mirab-balou et al. (2011) listed 18 genera and 59 species of Idolothripinae from China, but there continues to be no identification system available to these taxa. One genus that was listed by Mirab-balou, *Neosmerinthothrips* Bagnall, is not known from China. The record is an error because the single species involved, *Neosmerinthothrips brevicollis*, is known only from the Seychelles, and the name was presumably confused with a similarly named species of *Nesothrips*. Keys have been published to the species from China in a few genera, including *Bactrothrips*, *Gastrothrips*, *Meiothrips*, and *Ophthalmothrips* (Dang and Qiao 2012a, Cao et al. 2009, Dang and Qiao 2012b, Cao et al. 2010). Recently, Dang et al. (2013) reported some changes in nomenclature, including new synonyms, new combinations and new records from China.

### Methods and depositories

Descriptions and drawings are from slide-mounted specimens using Nikon Eclipse 80i & Leica DM4000B microscopes. Images were prepared with a Leica DM2500 using DIC illumination, and processed with Automontage and Photoshop software. Table 1 provides authority names and dates for all of the species discussed here, and full nomenclatural details and references for all Thysanoptera taxa are available in ThripsWiki 2013. Species that have not been studied here, and for which the information is from original descriptions, are indicated by an asterisk *. Slide-mounted specimens of all of these genera are available in the National Zoological Museum of China (NZMC), Institute of Zoology, Chinese Academy of Sciences, Beijing, China, and also the Australian National Insect Collection (ANIC), Canberra, Australia.

**Field work.** In China, netting and sweeping thrips living on plants and dead leaves has been the traditional collecting method. This method collects many specimens, but these are often damaged, the smaller species are not easily seen in a net, and almost no information on biology is produced. Precise field collecting methods are essential for good taxonomic research, and the best way to collect thrips in good condition is by carefully beating flowers, leaves, and dead hanging leaves and twigs, onto a white plastic plate. Thrips adhere to the plastic surface with their unique tarsal vesicle and can then be gathered into small vials of ethanol using a small brush. Furthermore, some thrips live in leaf litter, and samples of litter can be extracted through a Berlese or Tulgren Funnel into a collecting jar of ethanol. Detailed information on collecting thrips is available in ThripsWiki Website (ThripsWiki 2013).

**Microscope slides.** A major restraint on good taxonomic work on thrips is the large number of poorly-prepared specimens on microscope in many museum collections. In China, the method given by Han (1997) involves heating and macerating thrips in a strong NaOH solution (10%). However, this results in extensive damage and loss of colour to specimens. In contrast, Zhang et al. (2006) provided details of a method to make excellent slides, and details for slide preparation are also given in ThripsWiki (2013). Adults of the subfamily Idolothripinae have a wide range of body sizes, and many are large and dark. These dark thrips should be left in very weak NaOH solution (2%) for 12 hours or more at room temperature, but usually no more than 72 hours. It is hard work to make good slides, especially of these dark and large species, and this is one of the challenges when studying Idolothripinae.

### Key to genera of Idolothripinae from China

**Table:** 

1	Tube with prominent lateral setae (Fig. 54)	2
–	Tube smooth, without lateral setae, or lateral setae minute (Figs 11, 12)	6
2	Tube elongate, more than 10 times as long as basal width (Fig. 54)	3
–	Tube no more than 5 times as long as basal width	4
3	Head with projection in front of eyes much longer than broad, with two pairs of stout setae (Fig. 13); eyes distinctly prolonged ventrally (Fig. 13)	*Holurothrips*
–	Head with projection in front of eyes broader than long, with one pair of stout setae (Fig. 16); eyes equally developed on dorsal and ventral surfaces	*Meiothrips*
4	Maxillary stylets long, usually retracted to eyes, close together in middle of head	*Megalothrips*
–	Maxillary stylets short and wide apart, usually V-shaped	5
5	Pelta lateral lobes broadly joined to median lobe (Fig. 24); the distance between postocular setae less than half of head width behind eyes (Fig. 14); tergites II–VII each with two wing-retaining setae usually well developed; antennal segment III usually longer than head width across eyes	*Bactrothrips*
–	Pelta lateral lobes narrowly joined to, or separated from, median lobe (Figs 25, 26, 47); the distance between postocular setae about half of head width behind eyes; tergites II–VII of macropterae with anterior pair of wing-retaining setae small; antennal segment III much shorter than head width across eyes (Fig. 43)	*Megathrips*
6	Abdominal tergites III–V each with three pairs of sigmoid wing-retaining setae (Fig. 50)	*Mecynothrips*
–	Abdominal tegites III–V each with at most two pairs of sigmoid wing-retaining setae (Fig. 52)	7
7	Metathoracic sternopleural sutures absent	8
–	Metathoracic sternopleural sutures present	13
8	Eyes prolonged posteriorly on ventral surface of head (Figs 17, 41)	9
–	Eyes equally developed ventrally and dorsally	10
9	Antennal segment IV with 3 (rarely 2) sensoria; abdominal tergites II–VII each with one pair of sigmoid wing-retaining setae in macroptera	*Bolothrips*
–	Antennal segment IV with 4 sensoria; abdominal tergites II–VII usually each with two pairs of sigmoid wing-retaining setae	*Ophthalmothrips*
10	Female with inner margin of fore femur with row of about 4 tubercles at least (Figs 34, 35)	*Machatothrips*
–	Fore femur of female without tubercles on inner margin	11
11	Abdominal tegites III–V each with one pair of sigmoid wing-retaining setae (Fig. 52)	*Ethirothrips*
–	Abdominal tegites III–V each with two pairs of sigmoid wing-retaining setae	12
12	Pelta divided into three lobes (Fig. 31); mesothoracic spiracular area of male produced into prominent process (Fig. 36)	*Dinothrips*
–	Pelta not divided into three lobes (Figs 29, 30); mesothorax of male normal	*Elaphrothrips*
13	Metathoracic sternopleural sutures complete (Fig. 53); eyes distinctly prolonged ventrally	*Compsothrips*
–	Metathoracic sternopleural sutures incomplete; eyes usually not prolonged ventrally	14
14	Maxillary palp with a large stout terminal sensorium (Fig. 45)	*Allothrips*
–	Maxillary palp without stout terminal sensorium, or small	15
15	Maxillary stylets close together medially (Figs 42, 44)	16
–	Maxillary stylets wide apart, almost V-shaped (Figs 1, 2, 3, 40)	17
16	Antennal segment IV with 2 sensoria	*Phaulothrips*
–	Antennal segment IV with 3 sensoria	*Cryptothrips*
17	Antenna 7-segmented, segment VII with an incomplete suture	*Acallurothrips*
–	Antenna 8-segmented, but segments VII and VIII sometimes broadly joined (Fig. 46)	18
18	Antennal segment IV with 3 sensoria	*Gastrothrips*
–	Antennal segment IV with 4 sensoria	*Nesothrips*

### Acallurothrips

Bagnall

http://species-id.net/wiki/Acallurothrips

#### Remarks.

There are 22 species listed in this genus, of which five are recorded from China: *Acallurothrips tubullatus* from Guangdong (Wang and Tong 2008), and *Acallurothrips casuarinae*, *Acallurothrips hagai*, *Acallurothrips hanatanii* and *Acallurothrips nonakai* from Taiwan (Okajima 2006), of which paratypes were studied in ANIC.

#### Diagnosis.

Head usually broad; postocular setae usually longer than eye, and pointed at apex; stylets long and wide apart; antennae 7-segmented, VII and VIII usually joined with incomplete or complete suture, III with 2 sensoria, IV with 4; pronotum with 4 or 5 pairs of acute setae, sometimes anteroangular setae reduced; notopleural sutures incomplete or complete; basantra present, mesopraesternum reduced a small plate or absent; sternopleural sutures present; fore tarsal tooth present in both sexes; fore wings broad, usually without duplicated cilia; pelta irregular, usually eroded at posterior margin medially; tergites II–VII with 1 pair of wing-retaining setae; tube usually with sides convex, maximum width more than twice apical width; anal setae much shorter than tube.

#### Key to *Acallurothrips* species from China

**Table:** 

1	Tube longer, more than 1.3 times as long as the widest part (Fig. 4)	2
–	Tube broad, about as long as the widest part (Figs 5, 6)	3
2	Pronotal notopleural sutures incomplete (Fig. 1); accessory setae S2 on abdominal tergite IX well-developed	*Acallurothrips hanatanii*
–	Pronotal notopleural sutures complete; accessory setae S2 on abdominal tergite IX minute (Fig. 4)	*Acallurothrips nonakai*
3	Accessory setae S2 on abdominal tergite IX minute; postocular setae about as long as eyes (Fig. 3)	*Acallurothrips casuarinae*
–	Accessory setae S2 on abdominal tergite IX well-developed (Fig. 5); postocular setae much longer than eyes	4
4	Head broad, about 1.6 times as broad as long; postocular setae about 2.0 times as long as eyes	Acallurothrips tubullatus*
–	Head about 1.2 times as broad as long; postocular setae about 1.4 times as long as eyes (Fig. 2)	*Acallurothrips hagai*

**Figures 1–6.**
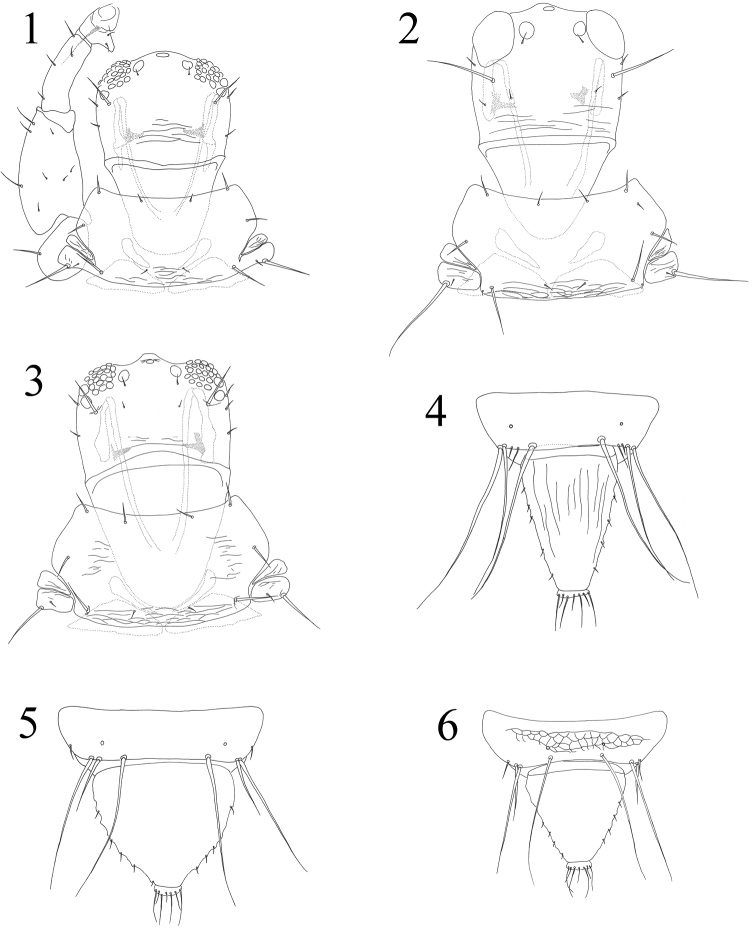
*Acallurothrips* spp. **1–3** Head & pronotum: **1** *Acallurothrips hanatanii* **2** *Acallurothrips hagai* **3** *Acallurothrips casuarinae* **4–6** Abdominal tergites IX–X: **4** *Acallurothrips nonakai* **5** *Acallurothrips hagai* **6** *Acallurothrips casuarinae*.

### Allothrips

Hood

http://species-id.net/wiki/Allothrips

#### Remarks.

There are 24 species listed in this genus, of which three are recorded in China: *Allothrips bicolor* from Hainan (Han 1997), *Allothrips discolor* and *Allothrips taiwanus* from Taiwan (Chen 1982, Okajima 1987). Species of this genus are usually wingless, and are distinguish from other Idolothripinae by the unusually large terminal sensorium on the maxillary palp (Fig. 45), and the 7-segmented antennae.

#### Diagnosis.

Head slightly longer than broad; dorsum of head usually with 3 or 4 pairs of well developed capitate setae; maxillary palp terminal sensorium stout, stylets long and wide apart; antennae 7-segmented, morphological VII and VIII completely fused, III with 2 sensoria, IV with 2; pronotal major setae usually expanded, notopleural sutures incomplete or complete; basantra present, mesopraesternum reduced; sternopleural sutures present; fore tarsal tooth usually absent in female, present in male; usually aptera; pelta broad, with obviously lateral lobes; tube straight sided, usually shorter than head; anal setae usually slightly longer than tube.

#### Key to *Allothrips* species from China

**Table:** 

1	Dorsal surface of body fully reticulated	Allothrips taiwanus*
–	Dorsal surface of body smooth or simply sculptured	2
2	Postocular cheek setae small, much shorter than postoculars	Allothrips discolor*
–	Postocular cheek setae well developed, length more than 0.5 of postocular setae, with apex expanded	*Allothrips bicolor*

### Bactrothrips

Karny

http://species-id.net/wiki/Bactrothrips

#### Remarks.

Currently there are 53 species listed in this genus, of which seven have been recorded from China: *Bactrothrips brevitubus*, *Bactrothrips elongatus*, *Bactrothrips flectoventris*, *Bactrothrips furvescrus*, *Bactrothrips honoris*, *Bactrothrips pictipes* and *Bactrothrips quadrituberculatus*. Dang and Qiao (2012a) provided an identification key to these seven species, based on morphological and DNA barcoding data. The genus *Bactrothrips* is closely related to *Megathrips*, and differs from *Megalothrips* in usually having shorter maxillary stylets.

#### Diagnosis.

Head much longer than width across eyes, usually prolonged in front of eyes; eyes usually equally developed ventrally and dorsally, sometimes obviously prolonged on ventral surface (*Bactrothrips flectoventris*); dorsum of head usually with 4 pairs of well-developed setae; stylets short and far apart, or long and close together; antennae 8-segmented, segment III with 2 sensoria, IV with 4; pronotum usually with 5 pairs of well-developed major setae, sometimes epimeral accessory setae also well-developed, notopleural sutures incomplete or complete; basantra present; mesopraesternum boat-shaped; metathoracic sternopleural sutures absent; wings usually fully developed with numerous duplicated cilia, sometimes apterous; fore tarsal tooth absent in both sexes; pelta broad, with two lobes; abdominal tergites II–VII each with 2 pairs of sigmoid wing-retaining setae; tergites V–VIII of male with or without lateral tubercles; tube surface with numerous fine setae; anal setae much shorter than tube.

### Bolothrips

Priesner

http://species-id.net/wiki/Bolothrips

#### Remarks.

There are 17 species listed in this genus, although none have previously been recorded from China. However, specimens from Northern China representing two unidentified species of this genus are available in NZMC.

#### Diagnosis.

Head usually longer than wide, projecting in front of eyes; eyes strongly prolonged ventrally; postocular setae well-developed; stylets V-shaped; antennae 8-segmented, segment III with 2 or 1 sensoria, IV with 3 or 2; pronotal major setae pointed or blunt, notopleural sutures complete; basantra present; mesopraesternum boat-shaped; metathoracic sternopleural sutures absent; wings, if present, with numerous duplicated cilia; fore tarsal tooth present in male; pelta broad, rounded triangular; abdominal tergites II–VII each with 1 pair of sigmoid wing-retaining setae in macroptera; tube surface smooth, without prominent setae; anal setae about as long as tube.

### Compsothrips

Reuter

http://species-id.net/wiki/Compsothrips

#### Remarks.

There are 27 species listed in this genus, of which three are recorded from China: *Compsothrips reticulates*, *Compsothrips sinensis* and *Compsothrips tenebronus* (Guo and Feng 2006, Pelikan 1961, Han and Cui 1991). Dang et al. (2013) transferred *Ophthalmothrips tenebronus* Han & Cui to *Compsothrips* as a new combination, and also placed *Cryptothrips furvus* Reyes from Philippines as a new synonym of that species. Pelikan (1961) described *Compsothrips sinensis* from Guangdong, China based on a single female specimen, and indicated that the holotype was deposited in NZMC, Institute of Zoology, Chinese Academy of Sciences. However, that specimen has not been found in NZMC, nor is it in the Pelikan collection in Slovakia, and it is possibly lost. From the original description, *Compsothrips sinensis* cannot be distinguished with *Compsothrips tenebronus*, and they may represent the same species, so *Compsothrips sinensis* is here excluded from the key below.

#### Diagnosis.

Head much longer than wide, projecting in front of eyes; eyes strongly prolonged ventrally; postocular setae well-developed, one pair of ocellar setae developed; stylets V-shaped; antennae 8-segmented, segment III with 2 or 1 sensoria, IV with 2, sensoria small; pronotal major setae expanded, notopleural sutures complete; basantra present; mesopraesternum boat-shaped; metathoracic sternopleural sutures complete; usually apterous; fore tarsal tooth present in both sexes; pelta broad, rounded triangular; abdominal tergites usually without sigmoid wing-retaining setae; tube surface smooth, without prominent setae; anal setae shorter or a little longer than tube.

#### Key to *Compsothrips* species from China

**Table:** 

1	Antennal segment III with one sensorium	Compsothrips reticulates*
–	Antennal segment III with two sensoria	*Compsothrips tenebronus*

### Cryptothrips

Uzel

http://species-id.net/wiki/Cryptothrips

#### Remarks.

There are 12 species listed in this genus, of which only one, *Cryptothrips nigripes*, is known from China, this Palaearctic species having been recorded from Inner Mongolia by Dang et al. (2013). Also *Cryptothrips sauteri* was described from Taiwan, but judging from the original description it was based on a single specimen that lacks antennae. This species cannot be recognized, and even its generic relationship remains unknown. The original specimen is not in the Senckenberg Museum, Frankfurt, where so many of Karny’s specimens are deposited, and is probably lost. The references in Mirab-balou (2011) are simply bibliographic quotations.

#### Diagnosis.

Head longer than wide; eyes equally developed ventrally and dorsally; postocular setae well-developed, ocellar setae usually small; stylets long and close together medially; antennae 8-segmented, segment III with 2 sensoria, IV with 3; pronotal major setae usually pointed or blunt, notopleural sutures complete; basantra present; mesopraesternum boat-shaped; metathoracic sternopleural sutures present; wings, if present, with duplicated cilia; fore tarsal tooth present in male, absent in female; pelta broad, with two slender lobes; abdominal tergites III–VI with 1 pair of sigmoid wing-retaining setae; tube surface smooth, without prominent setae; anal setae usually shorter than tube.

### Dinothrips

Bagnall

http://species-id.net/wiki/Dinothrips

#### Remarks.

This genus comprises six Asian species, of which four are recorded from southern China, *Dinothrips hainanensis*, *Dinothrips juglandis*, *Dinothrips spinosus* and *Dinothrips sumatrensis*. Species of this genus can be recognised by the pelta divided into three separate parts, and the males with the mesothoracic spiracular area curiously expanded into a prominent process (Mound and Palmer 1983). Species differ in the shape of this process in males, but females cannot be identified to species with any certainty. The spiracular process of males varies with body size within *Dinothrips spinosus*, and the shape also varies in slide-mounted specimens due to cover-slip pressure. As a result, it seems likely that *Dinothrips hainanensis* is the same species as *Dinothrips spinosus*. Here, the key to three species of *Dinothrips Dinothrips* from China excludes *Dinothrips hainanensis*.

#### Diagnosis.

Head much longer than wide, projecting slightly in front of eyes, cheeks with numbers of stout setae; eyes equally developed ventrally and dorsally; postocular setae well developed, interocellar setae usually developed; stylets V-shaped; antennae 8-segmented, segment III with 2 sensoria, IV with 4; pronotal major setae usually pointed or blunt, notopleural sutures complete; basantra present; mesothoracic spiracular area of male usually prolonged into prominent process; mesopraesternum boat-shaped; metathoracic sternopleural sutures absent; wings, if present, with duplicated cilia; fore tarsal tooth present in both sexes, a series tubercles present on inner margin of fore tibiae in large males; pelta divided into one large median lobe, 2 small lateral lobes; abdominal tergites III–VI with 2 pairs of sigmoid wing-retaining setae; tube surface smooth, without prominent setae; anal setae usually shorter than tube.

#### Key to *Dinothrips* species from China

**Table:** 

1	Antennal segment III largely yellow with brown apex	*Dinothrips juglandis*
–	Antennal segment III largely yellow but brown at apex and base	2
2	Antennal segment III short, about 3 times as long as apical brown part	*Dinothrips sumatrensis*
–	Antennal segment III elongate, more than 4 times as long as apical brown part	*Dinothrips spinosus*

### Elaphrothrips

Buffa

http://species-id.net/wiki/Elaphrothrips

#### Remarks.

Species of this genus can be found in all tropical countries, and 141 species are currently listed, with eight recorded from China: *Elaphrothrips denticollis*, *Elaphrothrips jacobsoni*, *Elaphrothrips greeni*, *Elaphrothrips spiniceps*, *Elaphrothrips fulmeki*, *Elaphrothrips insignis*, *Elaphrothrips malayensis* and *Elaphrothrips procer*. The last four are here newly recorded from China, but there are also several undescribed species represented in NZMC. The species *Elaphrothrips denticollis* is widespread in Southern China, and shares most characters with *Elaphrothrips malayensis*, especially fore tarsi elongate. There is no satisfactory differentiation between these two species, although Palmer and Mound (1978) distinguished *Elaphrothrips denticollis* from *Elaphrothrips malayensis* by antennal segments IV–V being uniformly brown or with the basal stem light brown. This is difficult to assess in many specimens that have been slightly bleached. There is a similar problem with *Elaphrothrips fulmeki* and *Elaphrothripsw malayensis* that are distinguished only by antennal segment VI with the basal 1/5 brown or pale. Currently, the identification of some *Elaphrothrips* species is not satisfactory.

#### Diagnosis.

Head much longer than wide, projecting in front of eyes, cheeks usually with numbers of stout setae; eyes equally developed ventrally and dorsally, or a little prolonged ventrally; postocular setae well-developed, interocellar setae well developed, and one pair of median dorsal setae usually developed; stylets short and V-shaped; antennae 8-segmented, segment III with 2 sensoria, IV with 4; pronotal major developed setae usually pointed or blunt, notopleural sutures complete or nearly complete; basantra present; mesopraesternum boat-shaped; metathoracic sternopleural sutures absent; wings, if present, with duplicated cilia; fore tarsal tooth present in male, absent in female, fore femur of large males usually with sickle-shaped seta on external apical margin; pelta broad, two lateral lobes broadly joined with middle one; abdominal tergites III–VI with 2 pairs of sigmoid wing-retaining setae; tube surface smooth, without prominent setae; anal setae usually shorter than tube.

#### Key to *Elaphrothrips* species from China

**Table:** 

1	Head with cheek setae dark	2
–	Head with cheek setae yellow or pale	3
2	Head only slightly produced in front of eyes, length about 1/8 as long as width; inner margin of male fore femora with tubercles (Fig. 37); pelta broadly joined to lateral wings (Fig. 29)	*Elaphrothrips insignis*
–	Head strongly produced in front of eyes, length about 1/2 as long as width; inner margin of fore femora without tubercles in both sexes; pelta narrowly jointed to lateral wings or separated	Elaphrothrips jacobsoni*
3	Head process in front of eyes short, width 3–5 times its length; tibiae uniformly dark brown	4
–	Head process in front of eyes long, width 1–3 times its length; tibiae sometimes pale in the apical half	5
4	Head produced process very short, width about 5 times as long as length (Fig. 22); antennal segment III stout and short, about twice as long as apical brown part; pelta narrowly jointed with lateral wings, joint slender	*Elaphrothrips spiniceps*
–	Head produced process short, width about 3 times as long as length; antennal segment III more than 4 times as long as apical brown part; pelta narrowly jointed with lateral wings, joint short (Fig. 30)	*Elaphrothrips procer*
5	Tibiae largely yellow, with sub-basal 1/3 brown (head produced process about as long as width) (Fig. 23)	*Elaphrothrips greeni*
–	Mid tibiae uniformly brown at least	6
6	Antennal segments IV–V uniformly brown, sometimes a little shallow at base; fore tarsal elongate, about 3 times as long as width (Fig. 38)	*Elaphrothrips denticollis*
–	Antennal segments IV–V brown with basal pedial pale; fore tarsal various	7
7	Antennal segment III longer than IV; VI brown with basal 1/5 yellow (Fig. 32)	*Elaphrothrips fulmeki*
–	Antennal segment III as long as IV; VI uniform brown	*Elaphrothrips malayensis*

### Ethirothrips

Karny

http://species-id.net/wiki/Ethirothrips

#### Remarks.

This genus currently comprises 37 species, of which eight are recorded from China: *Ethirothrips brevis*, *Ethirothrips indicus*, *Ethirothrips obscurus*, *Ethirothrips chui* and *Ethirothrips virgulae* from Taiwan, *Ethirothrips longisetis* and *Ethirothrips vitreipennis* from Hainan, and *Ethirothrips stenomelas* from Guangdong and Hainan. However, *Ethirothrips virgulae* is here newly recorded from mainland China at Guangxi. In this study, type-specimens of two species of Chen (Taiwan Agricultural Research Institute, Taichung) were checked, but unfortunately they are so poor that most characters could not be studied.

#### Diagnosis.

Head usually longer than width across eyes; eyes small, equally developed ventrally and dorsally; postocular setae well-developed; stylets long, V-shaped or sub-parallel; antennae 8-segmented, segment III with 2 sensoria, IV 4; pronotal major setae pointed or blunt, notopleural sutures complete; basantra present; mesopraesternum boat-shaped; metathoracic sternopleural sutures absent; wings, if present, with numerous duplicated cilia; fore tarsal tooth present in male; pelta broad, with two broad lateral lobes; abdominal tergites II–VII each with 1 pair of sigmoid wing-retaining setae in macroptera; tube surface smooth, without prominent setae; anal setae shorter than tube.

#### Key to *Ethirothrips* species from China

**Table:** 

1	Antennal segment IV with five sensoria	*Ethirothrips stenomelas*
–	Antennal segment IV with four sensoria	2
2	Postocellar setae elongate, longer than diameter of posterior ocelli (Figs 7, 8)	3
–	Postocellar setae reduced, much shorter than diameter of posterior ocelli (Fig. 9)	4
3	Antennal segments IV–VIII uniformly brown, at least IV darker than III; abdominal segment IX with posteromarginal setae shorter than tube (Fig. 11)	*Ethirothrips indicus*
–	Antennal segment IV as yellow as III, V–VIII brown; abdominal segment IX with posteromarginal setae longer than tube (Fig. 12)	*Ethirothrips obscurus*
4	Major setae blunt at apex	5
–	Major setae acute at apex	6
5	Metanotum without campaniform sensilla	*Ethirothrips chui*
–	Metanotum with pair of campaniform sensilla (Fig. 10)	*Ethirothrips brevis*
6	Major setae well developed, postocular setae about 230 microns	Ethirothrips longisetis*
–	Major setae relatively shorter, postocular setae about 100 microns	7
7	Body uniformly brown to dark brown	*Ethirothrips virgulae*
–	Body bicolored, largely yellow except brown tube	*Ethirothrips vitreipennis*

**Figures 7–12.**
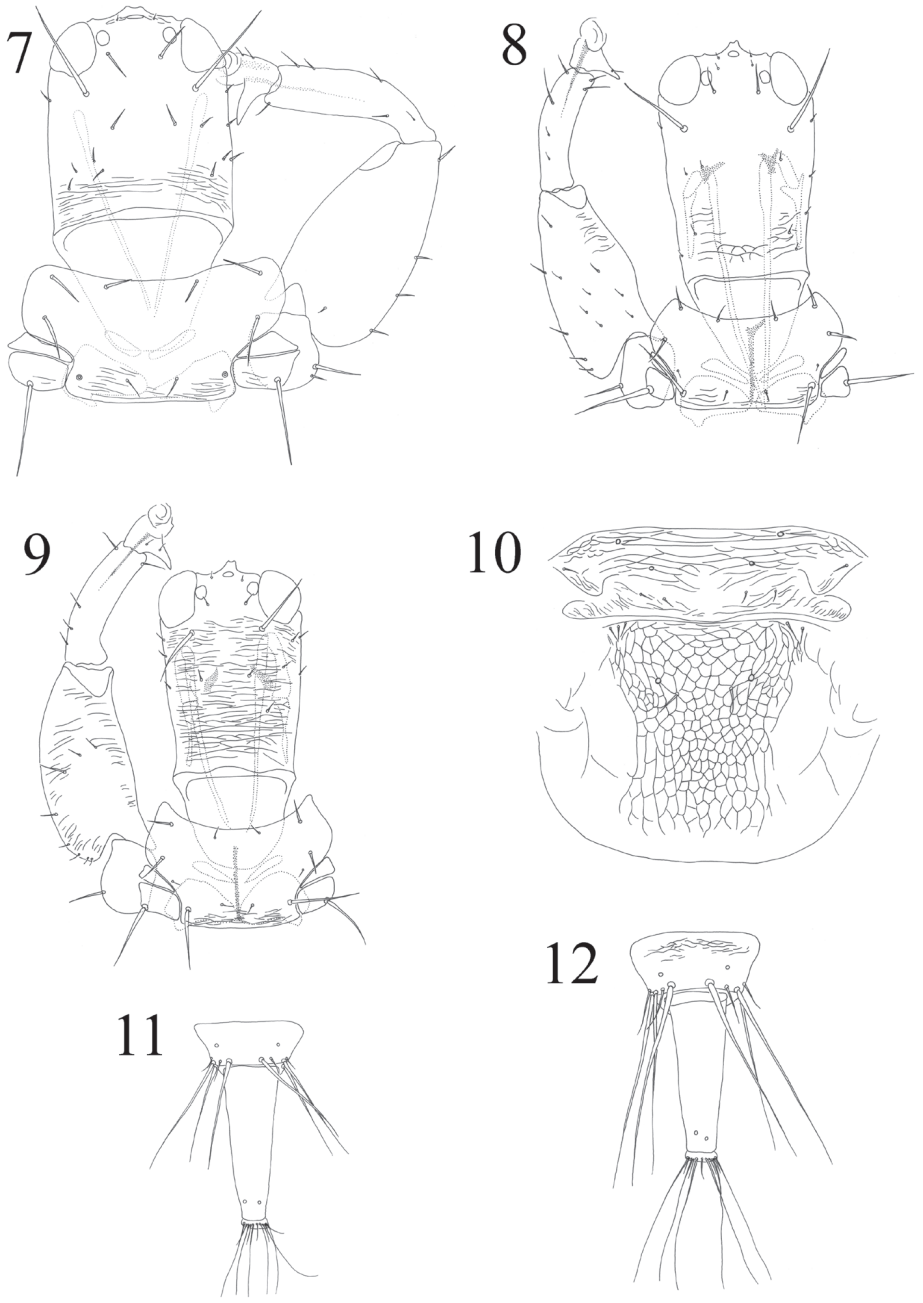
*Ethirothrips* spp. **7–9** Head, pronotum & foreleg: **7** *Ethirothrips indicus* **8** *Ethirothrips obscurus* **9** *Ethirothrips vitreipennis* Mesonotum & metanotum: **10** *Ethirothrips brevis* **11–12** Female abdominal tergites IX–X: **11** *Ethirothrips indicus* **12** *Ethirothrips obscurus*.

**Figures 13–19.**
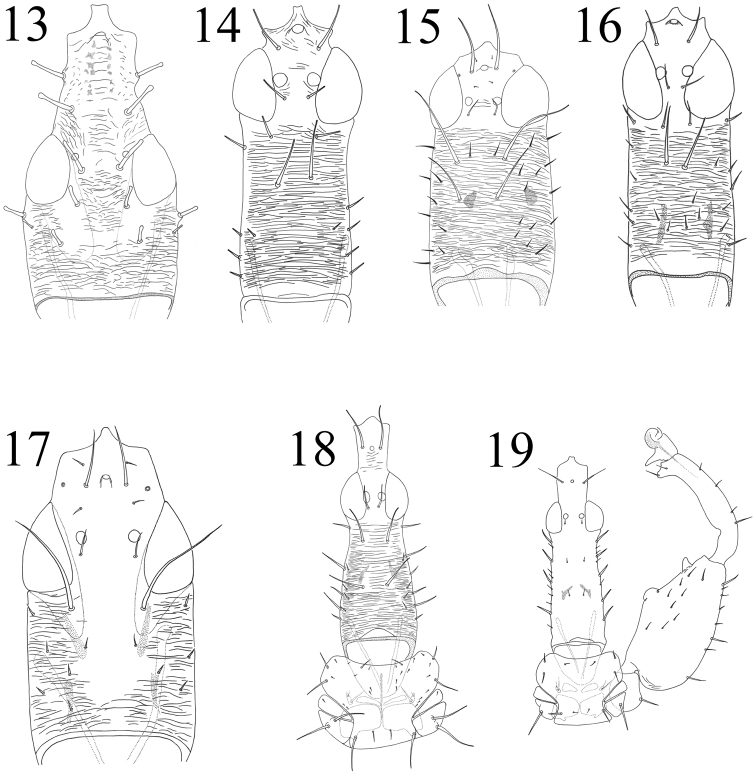
**13–17** Idolothripinae head: **13** *Holurothrips* **14** *Bactrothrips* **15** *Elaphrothrips* **16** *Meiothrips* **17** *Ophthalmothrips* **18** *Mecynothrips simplex*, head & pronotum **19** *Mecynothrips pugilator*, head, pronotum & foreleg.

### Gastrothrips

Hood

http://species-id.net/wiki/Gastrothrips

#### Remarks.

There are 38 species listed in this genus, of which five are recorded from China: *Gastrothrips fuscatus*, *Gastrothrips fulviceps*, *Gastrothrips mongolicus*, *Gastrothrips eurypelta*, also *Gastrothrips monticola* that was described from Japan by Okajima (2006) but is here newly recorded from Inner Mongolia, China. One species, *Gastrothrips fulviceps*, was described by Hood (1937) from Peru with the head and antennae colored distinctively, and this species was recorded from Taiwan by Kudo (1974) as *Nesothrips fulviceps* (Hood). *Gastrothrips mongolicus* which was described by Pelikan (1965) based on two females, was first recorded from China by Cao et al. (2009) together with a new species *Gastrothrips eurypelta*. However, Cao et al. (2009) indicated in a key that the postocular setae of *Gastrothrips mongolicus* are pointed at the apex, and was the only difference given from *Gastrothrips eurypelta*. However, the description of *Gastrothrips mongolicus* stated that these setae are blunt, also the pronotal major setae. Furthermore, although the description of *Gastrothrips eurypelta* did not mention the ventral shape of the eyes, the illustration indicated the eyes are slightly prolonged ventrally, as is illustrated for *Gastrothrips mongolicus* by Okajima (2006). Thus *Gastrothrips eurypelta* is probably a synonym of *Gastrothrips mongolicus*, and is excluded from the key below.

#### Diagnosis.

Head usually as long as broad, or a little longer; eyes normal, usually equally developed ventrally and dorsally; postocular setae well-developed; stylets usually V-shaped; antennae 8-segmented, segment III with 1 or 2 sensoria, IV with 3; pronotal major setae pointed or blunt, notopleural sutures complete or incomplete; basantra present; mesopraesternum boat-shaped; metathoracic sternopleural sutures present; wings, if present, with or without numerous duplicated cilia; fore tarsal tooth present in male; pelta triangular, or with two broad lateral lobes; abdominal tergites II–VII each with 1 pair of sigmoid wing-retaining setae in macroptera; tube surface smooth, without prominent setae; anal setae shorter than tube.

#### Key to *Gastrothrips* species from China

**Table:** 

1	Antennal segment III with one sensorium	2
–	Antennal segment III with two sensoria	3
2	Head uniformly brown, concolorous with thorax; antennal segments uniform brown, concolorous with head, except III yellow; postoculars and pronotal major setae nobbed at apex	Gastrothrips fuscatus*
–	Head bicolored, the front yellow, sides and basal part brown; antennal segments I–VI largely golden yellow; postoculars and pronotal major setae pointed at apex	Gastrothrips fulviceps*
3	Postoculars and pronotal major setae pointed at apex	*Gastrothrips monticola*
–	Postoculars and pronotal major setae blunt at apex	*Gastrothrips mongolicus*

### Holurothrips

Bagnall

http://species-id.net/wiki/Holurothrips

#### Remarks.

There are four species listed in this genus. Only *Holurothrips morikawai* is recorded from China, and this species is here newly recorded from Taiwan.

#### Diagnosis.

Head much longer than broad, with elongate projection in front of eyes; eyes obviously prolonged ventrally; 2 pairs of postoculars, 2 pairs of interocellars and 1 pair of postocellars well developed; cheeks with 1 pair of stout setae; stylets V- or U-shaped; antennae 8-segmented, segment III with 2 sensoria, IV with 2 or 4; pronotal with 5 pairs of major developed setae, anteroangulars close to midlaterals, notopleural sutures complete; basantra present; mesopraesternum boat-shaped; metathoracic sternopleural sutures absent; wings, if well developed, with duplicated cilia; fore tarsal tooth absent in both sexes, femur with a few stout setae; pelta wide, with two broad lobes; abdominal tergites II–VII each with 2 or 3 pairs of sigmoid wing-retaining setae; tube elongate, with prominent lateral setae; anal setae much shorter than tube.

### Machatothrips

Bagnall

http://species-id.net/wiki/Machatothrips

#### Remarks.

Of the 14 species included in this genus, three are recorded from China: *Machatothrips antennatus*, *Machatothrips artocarpi* and *Machatothrips celosia*. Several specimens of *Machatothrips antennatus* and *Machatothrips artocarpi* have been studied in ANIC, and these were identified from the types. The third species, *Machatothrips celosia*, was described from Taiwan and is added to the key below based on the key to 14 species by Palmer and Mound (1978).

#### Diagnosis.

Head much longer than broad; eyes normal; 1 pair of postoculars well-developed, also 1 pair of interocellars and 1 pair of vertex setae; stylets V-shaped; antennae 8-segmented, segment III with 2 sensoria, IV with 4; pronotum usually with 5 pairs of major setae, notopleural sutures complete; basantra present; mesopraesternum boat-shaped; metathoracic sternopleural sutures absent; fore wings with duplicated cilia; fore tarsal tooth present in both sexes; females with fore femur bearing a row of tubercles on inner margin; pelta broadly triangular; abdominal tergites II–VII each with 1 pair of sigmoid wing-retaining setae; tube longer than head, without prominent lateral setae; anal setae shorter than tube.

#### Key to *Machatothrips* species from China

**Table:** 

1	Pronotal anteromarginal setae longer than anteroangulars	Machatothrips celosia*
–	Pronotal anteromarginal setae shorter than anteroangulars	2
2	Postocular setae II minute, much shorter than pair I (Fig. 20); pronotal anterior margin with 3–5 pairs of stout setae laterally, shorter than anteromarginal setae (Fig. 20); inner margin of fore femora with more than 10 small series tubercles in female (Fig. 34)	*Machatothrips antennatus*
–	Postocular setae II developed, about 1/3 as long as pair I (Fig. 21); pronotal anterior margin without setae except anteromarginal setae (Fig. 21); inner margin of fore femora with 4–6 stout teeth in different size in female (Fig. 35)	*Machatothrips artocarpi*

**Figures 20–23.**
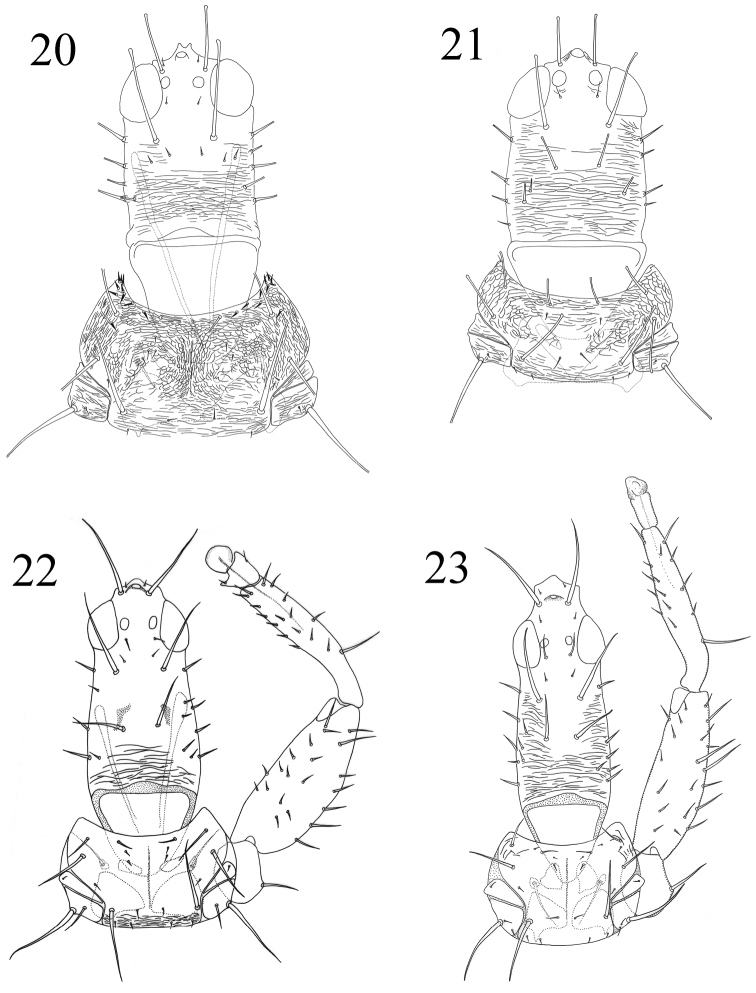
**20–21** *Machatothrips*, head & pronotum: **20** *Machatothrips antennatus* **21** *Machatothrips artocarpi* **22–23** *Elaphrothrips*, head, pronotum & foreleg: **22** *Elaphrothrips spiniceps* **23** *Elaphrothrips greeni*.

**Figures 24–31.**
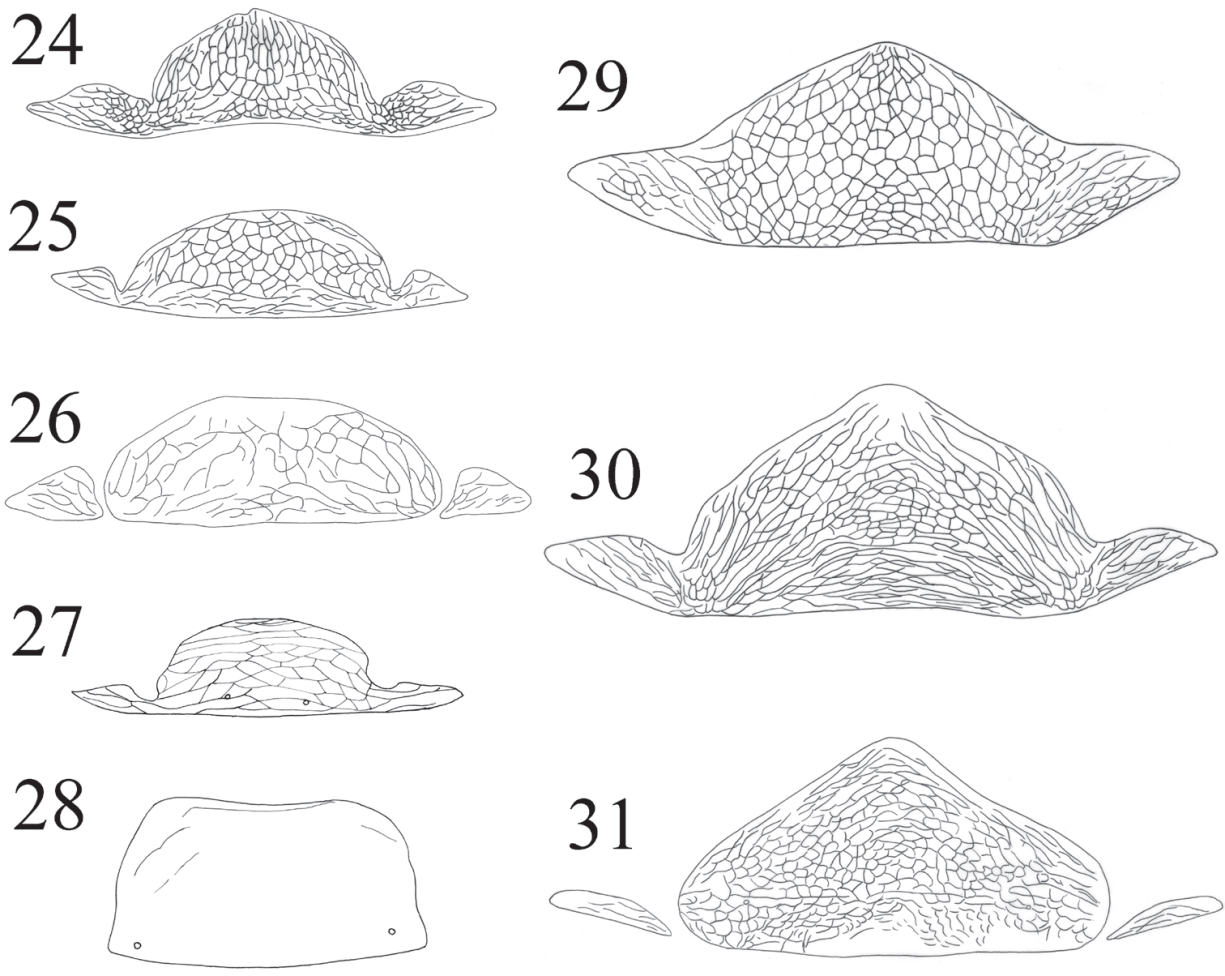
Idolothripinae Pelta: **24** *Bactrothrips* sp **25–26** *Megathrips lativentris* **27** *Nesothrips brevicollis* **28** *Nesothrips peltatus* **29** *Elaphrothrips insignis* **30** *Elaphrothrips procer* **31** *Dinothrips* sp.

**Figures 32–39.**
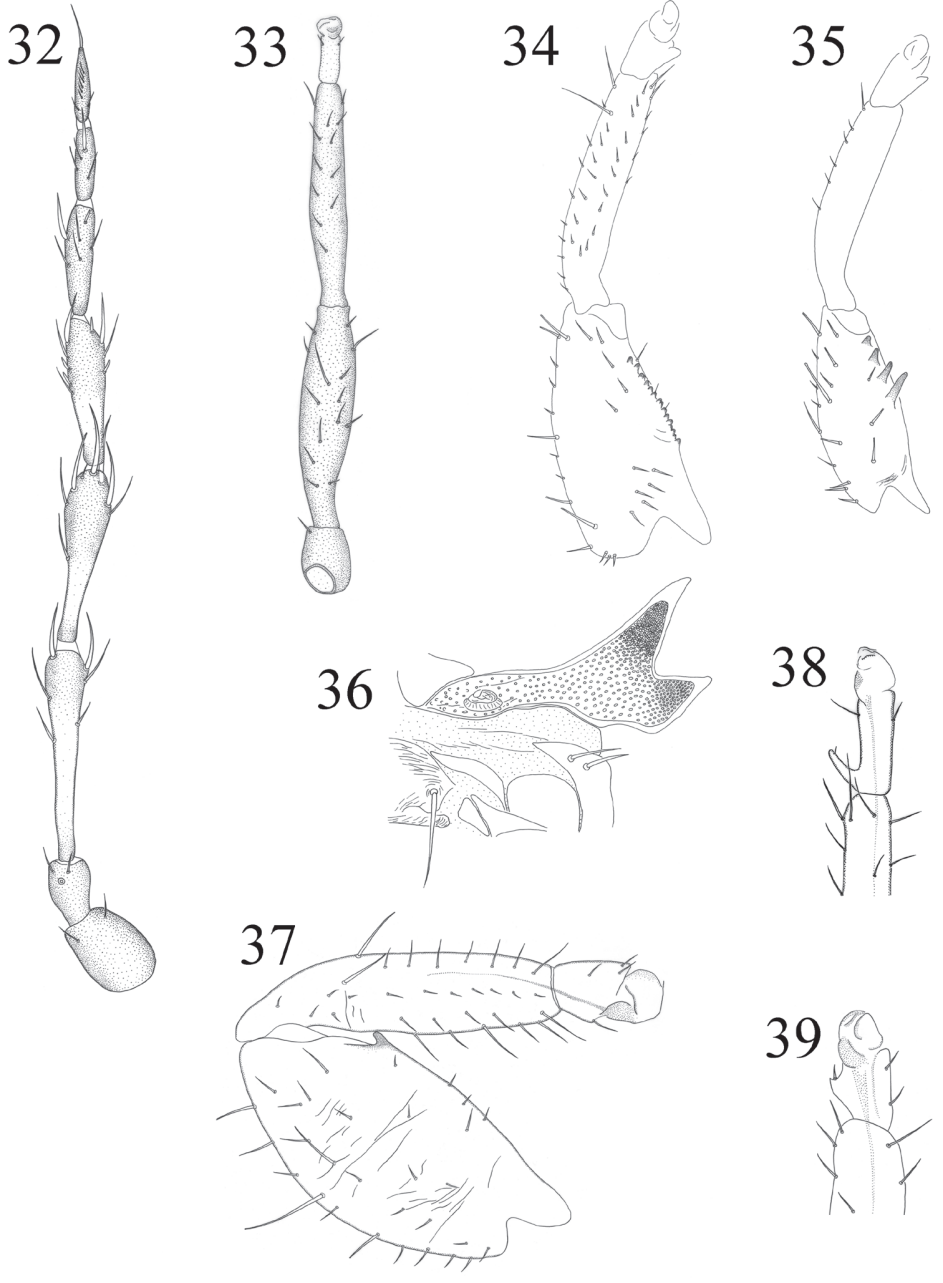
**32–33** *Elaphrothrips fulmeki*: **32** antennae **33** mid-leg **34–35** *Machatothrips* foreleg: **34** *Machatothrips antennatus* **35** *Machatothrips artocarpi* **36** *Dinothrips sumatrensis*, male mesothoracic spiracular process **37** *Elaphrothrips insignis*, male foreleg **38** *Elaphrothrips denticollis*, male fore tarsi **39** *Ophthalmothrips miscanthicola*, female fore tarsal tooth.

### Mecynothrips

Bagnall

http://species-id.net/wiki/Mecynothrips

#### Remarks.

The 14 species included in this genus involve some of the largest Thysanoptera, and in the proventriculus of adults there is a prominent basket-like structure that is probably involved in crushing the fungus spores on which these species feed (Tree et al. 2010). A similar structure also occurs in species of *Elaphrothrips*. Four species are recorded from China: *Mecynothrips kanoi*, *Mecynothrips pugilator*, *Mecynothrips simplex* and *Mecynothrips taiwanus*, of which *Mecynothrips simplex* is here newly recorded from China based on four females and eight males from Yunnan and Hainan Provinces. Okajima (1979) described *Mecynothrips taiwanus* from Taiwan, and this can be distinguished from *Mecynothrips pugilator* by having a longer preocular projection from base of antennal segment I to anterior margin of eyes about 1.5 times as long as wide, whereas in *Mecynothrips pugilator* this is about as long as wide. The species *Mecynothrips kanoi* was described from Taiwan, but the depositary of the syntypes is unknown, and no useful characters can be taken from the simple original description. Therefore, *Mecynothrips kanoi* is excluded in the following key to Chinese species of *Mecynothrips*.

#### Diagnosis.

Head much longer than broad, with prominent projection in front of eyes; eyes normal; 2 pairs of postoculars developed, also 1 pair of anterocellars well-developed, and 1 pair of postocellars; stylets short, V-shaped; antennae 8-segmented, segment III with 2 sensoria, IV with 4; pronotal major setae pointed or blunt, notopleural sutures usually complete, often incomplete; basantra present; mesopraesternum developed; metathoracic sternopleural sutures absent; fore wings with duplicated cilia; fore tarsal tooth present in male, absent in female, fore tibiae sometimes with seta-bearing apical tubercle in male, fore femur with a tumor or tubercles on inner margin in large male; pelta broad, with two prominent lateral lobes; abdominal tergites II–VII each with 2 or 3 pairs of sigmoid wing-retaining setae; tube smooth, without prominent lateral setae; anal setae shorter than tube.

#### Key to *Mecynothrips* species from China

**Table:** 

1	Pronotal epimeral accessory setae well-developed, as long as epimeral setae (Fig. 18); two pairs of postocular setae developed, as long as or longer than interocellar setae (Fig. 18)	*Mecynothrips simplex*
–	Pronotal epimeral accessory setae minute (Fig. 19); two pairs of postocular setae minute, much shorter than interocellar setae (Fig. 19)	2
2	Preocular projection shorter, about as long as wide (Fig. 19)	*Mecynothrips pugilator*
–	Preocular projection longer, about 1.5 times as long as wide	Mecynothrips taiwanus*

### Megalothrips

Uzel

http://species-id.net/wiki/Megalothrips

#### Remarks.

Of the eight species included in this genus, only *Megalothrips roundus* is recorded from China. The significance of four genera in the subtribe Idolothripina, *Bactrothrips*, *Megalothrips*, *Megathrips*, and *Meiothrips*, remains problematic (Dang and Qiao 2012a), and further studies are needed on the inter-relationships between the Holarctic and tropical faunas. The species of *Megalothrips* are identified by their remarkably elongate stylets which are close together medially, but they share other character states with *Bactrothrips* and *Megathrips* species.

#### Diagnosis.

Head much longer than broad, without prominent projection in front of eyes; eyes small; 1 pair of postoculars short, 1 pair of interocellars well-developed, and 1 pair of vertex setae usually longer than postoculars; stylets elongate, reaching eyes and close together; antennae 8-segmented, segment III with 2 sensoria, IV with 4; pronotal setae vary, anteroangulars close to midlaterals, notopleural sutures reduced; basantra present; mesopraesternum developed; metathoracic sternopleural sutures absent; fore wings with duplicated cilia; fore tarsal tooth absent in both sexes; pelta hat-shaped, with two slender lateral lobes narrowly fused; abdominal tergites II–VII each with 2 pairs of sigmoid wing-retaining setae; tergite VI with a pair of tubercles laterally in male; tube with prominent lateral setae; anal setae shorter than tube.

### Megathrips

Targioni-Tozzetti

http://species-id.net/wiki/Megathrips

#### Remarks.

There are seven species listed in this genus, of which two, *Megathrips lativentris* and *Megathrips antennatus*, are recorded from northern China. The original description of *Megathrips antennatus* states that it differs in having the pelta divided into three parts in contrast to the European species *Megathrips lativentris* that has the two lobes narrowly joined to the median one (Figs 25, 26). However, we studied the types of *Megathrips antennatus*, and in one paratype the left lobe of the pelta is separated from median one but the right lobe is narrowly joined to the median one. Furthermore, a female and a male from England studied in ANIC show that the lateral lobes of pelta slightly joined to the median one, or close to separate (Fig. 47). As a result, *Megathrips antennatus* Guo, Feng & Duan (2005) is here considered to be a new synonym of *Megathrips lativentris* (Heeger). Mound and Palmer (1983) indicated that *Megathrips* could be distinguished from *Bactrothrips* only by the slightly larger head and more deeply retracted stylets. However, in China, *Megathrips* and *Bactrothrips* species are similar in having the stylets short and V-shaped, but the lateral lobes of the pelta are broadly fused to the median lobe in *Bactrothrips* species whereas these lateral lobes are separate or narrowly joined in *Megathrips* species (Dang and Qiao 2012a).

#### Diagnosis.

Head usually longer than width across eyes, slightly prolonged in front of eyes; eyes normal; interocellar, postocellar, postocular and mid-dorsal setae usually well-developed; stylets far apart; antennae 8-segmented, segment III shorter than head width across eyes, segment III with 2 sensoria, IV with 4; pronotal major setae usually well developed, anteroangulars close to midlaterals, notopleural sutures incomplete; basantra present; mesopraesternum boat-shaped; metathoracic sternopleural sutures absent; wings usually fully developed with numerous duplicated cilia; fore tarsal tooth absent in both sexes; pelta always broad, lateral lobes narrowly joined to median major lobe, or separated; abdominal tergites II–VII each with 2 pairs of sigmoid wing-retaining setae in macroptera; tergite VI with a pair of long lateral tubercles in male, tubercles on VIII small; tube usually shorter than head, with numerous lateral setae; anal setae shorter than tube.

**Figures 40–46.**
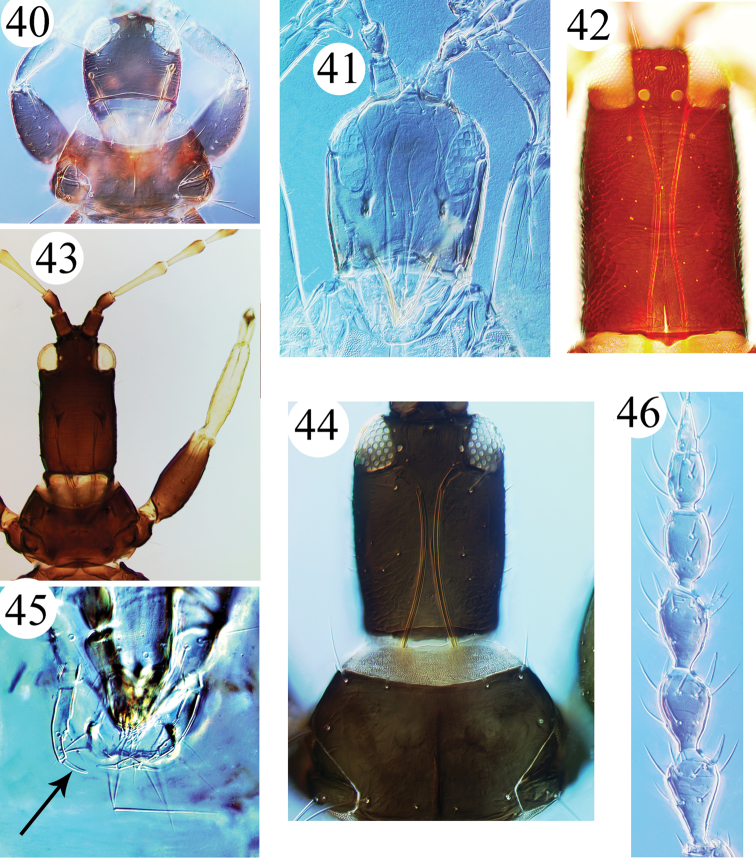
Idolothripinae genera. **40** *Gastrothrips*, head, pronotum & foreleg **41** *Bolothrips*, ventral view of head **42** *Phaulothrips*, head **43** *Megathrips*, head, pronotum & foreleg **44** *Cryptothrips*, head & pronotum **45** *Allothrips*, maxillary palps with stout terminal sensoria **46** *Gastrothrips*, antennal segments III–VIII.

**Figures 47–54.**
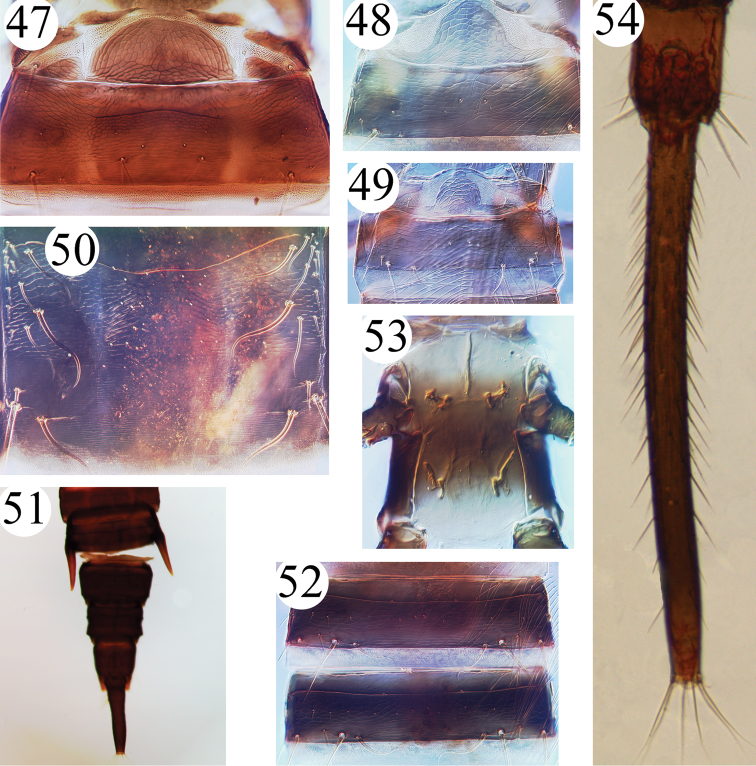
Idolothripinae genera. **47** *Megathrips lativentris*, pelta **48** *Ethriothrips*, pelta **49** *Gastrothrips*, pelta **50** *Mecynothrips*, abdominal tergite IV **51** *Megathrips*, male abdominal tergites VI–X **52** *Ethirothrips*, abdominal tergites IV–V **53** *Compsothrips*, ventral view of thorax **54** *Meiothrips*, male abdominal tergites IX–X.

### Meiothrips

Priesner

http://species-id.net/wiki/Meiothrips

#### Remarks.

Dang and Qiao (2012b) provided a key to the five known species of *Meiothrips*, of which three are recorded from China: *Meiothrips fuscicrus*, *Meiothrips menoni* and *Meiothrips nepalensis*. Moreover, *Meiothrips baishanzuensis* Duan & Li from Henan Province was synonymised with *Bactrothrips brevitubus* Takahashi by Dang and Qiao (2012a).

#### Diagnosis.

Head much longer than width across eyes, prolonged in front of eyes, usually shorter than broad except in one species (*Meiothrips kurosawai*) about twice as long as broad; eyes normal or clearly prolonged on ventral surface; interocellar, postocellar, postocular, mid-dorsal and posterior-dorsal setae usually well-developed, sometimes small; stylets short and far apart; antennae 8-segmented, very slender, segment III usually more than twice head width across eyes, segment III with 2 sensoria, IV with 4; pronotal major setae usually well developed, sometimes aa small and epimeral accessory setae always minute, notopleural sutures incomplete; basantra present; mesopraesternum boat-shaped; metathoracic sternopleural sutures absent; wings usually fully developed with or without numerous duplicated cilia; fore tarsal tooth absent in both sexes, femora with several spine-setae; pelta always broad, lateral lobes broadly joined to median lobe; abdominal tergites II–VII each with 2 pairs of sigmoid wing-retaining setae; male tergites V–VIII without lateral tubercles; tube much longer than head, with numerous lateral setae, sometimes with 2 rows of stout tubercles and many large and small tubercles or denticles on dorsal surface; anal setae much shorter than tube.

### Nesothrips

Kirkaldy

http://species-id.net/wiki/Nesothrips

#### Remarks.

There are 28 species listed in this genus, of which three are recorded from China: *Nesothrips brevicollis*, *Nesothrips lativentris* and *Nesothrips peltatus*. A further species, *Nesothrips atropoda* Duan et al. (1998) from Henan Province, was treated by Dang et al. (2013) as a synonym of the widespread Asian species *Nesothrips brevicollis*.

#### Diagnosis.

Head various, usually wider than long; eyes normal, sometimes prolonged ventrally; 1 pair of postoculars well-developed, sometimes postocellar setae elongate; stylets V-shaped; antennae 8-segmented, segment III with 2 sensoria, IV with 4; pronotal major setae pointed or slightly blunt, notopleural sutures complete; basantra present; mesopraesternum developed; metathoracic sternopleural sutures present; fore wings, if well developed, with duplicated cilia; fore tarsal tooth present in male, absent in female; pelta broadly hat-shaped; abdominal tergites II–VII each with 1 pair of sigmoid wing-retaining setae in macroptera; tube smooth without lateral setae; anal setae usually slightly shorter than tube.

#### Key to *Nesothrips* species from China

**Table:** 

1	Head clearly longer than wide	*Nesothrips lativentris*
–	Head about as long as width	2
2	Pelta rectangle, without lateral wings (Fig. 28)	*Nesothrips peltatus*
–	Pelta median rounded, with slender lateral wings (Fig. 27)	*Nesothrips brevicollis*

### Ophthalmothrips

Hood

http://species-id.net/wiki/Ophthalmothrips

#### Remarks.

Of the 11 species included in this genus, four are recorded from China: *Ophthalmothrips formosanus*, *Ophthalmothrips longiceps*, *Ophthalmothrips miscanthicola* and *Ophthalmothrips yunnanensis*. This last species was based on one female and three males, and the description indicates that it is unusual in having the tergal wing-retaining setae small and straight in macropterae (Cao et al. 2010). *Ophthalmothrips formosanus* is here newly recorded from mainland China, in Henan Province.

#### Diagnosis.

Head longer than broad, projecting in front of eyes; eyes distinctly prolonged ventrally; 1 pair of postoculars well developed, 1 pair of interocellar setae elongate; stylets short, V-shaped; antennae 8-segmented, segment III with 2 sensoria, IV with 2 or 4; pronotum usually with 4 pairs of major setae, anteromarginals short, notopleural sutures complete; basantra present; mesopraesternum developed; metathoracic sternopleural sutures absent; fore wings, if well developed, with duplicated cilia; fore tarsal tooth present or absent; pelta broadly triangular; abdominal tergites II–VII usually each with 2 pairs of sigmoid wing-retaining setae; tube smooth without lateral setae; anal setae various.

#### Key to *Ophthalmothrips* species from China

**Table:** 

1	Abdominal segments with wing-retaining setae small and straight in macroptera	Ophthalmothrips yunnanensis*
–	Abdominal segments with wing-retaining setae well developed and sigmoidal	2
2	Fore tarsal tooth present in both sexes (Fig. 39)	*Ophthalmothrips miscanthicola*
–	Fore tarsal tooth absent in both sexes	3
3	Postocular setae shorter than interocellar setae	*Ophthalmothrips longiceps*
–	Postocular setae longer than interocellar setae (Fig. 17)	*Ophthalmothrips formosanus*

### Phaulothrips

Hood

http://species-id.net/wiki/Phaulothrips

#### Remarks.

There are 20 species listed in the genus, of which 16 are known only from Australia. *Phaulothrips solifer*, described from Taiwan, is the only member of the genus known from China. A paratype female and male on loan from Japan have been studied here.

#### Diagnosis.

Head much longer than broad; eyes normal, sometimes prolonged dorsally; 1 pair of postoculars well developed, close together, anterocellar setae usually elongate; cheeks with 1 pair of stout setae just behind eye; stylets long, close together medially; antennae 8-segmented, segments III–IV each with 2 sensoria; pronotal major setae pointed or slightly blunt, notopleural sutures complete; basantra present; mesopraesternum developed; metathoracic sternopleural sutures present; fore wings, if well-developed, with duplicated cilia; fore tarsal tooth present in both sexes; pelta broad, with two slender lateral lobes; abdominal tergites II–VII each with 1 pair of sigmoid wing-retaining setae in macroptera; tube smooth without lateral setae; anal setae shorter than tube.

## Supplementary Material

XML Treatment for Acallurothrips

XML Treatment for Allothrips

XML Treatment for Bactrothrips

XML Treatment for Bolothrips

XML Treatment for Compsothrips

XML Treatment for Cryptothrips

XML Treatment for Dinothrips

XML Treatment for Elaphrothrips

XML Treatment for Ethirothrips

XML Treatment for Gastrothrips

XML Treatment for Holurothrips

XML Treatment for Machatothrips

XML Treatment for Mecynothrips

XML Treatment for Megalothrips

XML Treatment for Megathrips

XML Treatment for Meiothrips

XML Treatment for Nesothrips

XML Treatment for Ophthalmothrips

XML Treatment for Phaulothrips
